# Effects of moderate vs. high iso-inertial loads on power, velocity, work and hamstring contractile function after flywheel resistance exercise

**DOI:** 10.1371/journal.pone.0211700

**Published:** 2019-02-07

**Authors:** Francisco Piqueras-Sanchiz, Saúl Martín-Rodríguez, Luis Manuel Martínez-Aranda, Thiago Ribeiro Lopes, Javier Raya-González, Óscar García-García, Fábio Yuzo Nakamura

**Affiliations:** 1 Faculty of Health Sciences, Universidad de Valencia, Valencia, Spain; 2 Research Institute of Biomedical and Health Sciences (IUIBS), University of Las Palmas de Gran Canaria, Las Palmas de Gran Canaria, Spain; 3 Neuroscience of Human Movement Research Group, Faculty of Sport, Catholic University San Antonio, Murcia (UCAM), Spain; 4 Olympic Centre of Training and Research, São Paulo, SP, Brazil; 5 São Paulo Association for Medicine Development, São Paulo, SP, Brazil; 6 Faculty of Health Sciences, Isabel I University, Burgos, Spain; 7 Lab of Performance, Fitness and Wellness, University of Vigo, Pontevedra, Spain; 8 The College of Healthcare Sciences, James Cook University, Townsville, Australia; 9 Department of Medicine and Aging Sciences, University of Chieti, Pescara, Italy; Universita degli Studi di Roma 'Foro Italico', ITALY

## Abstract

Flywheel iso-inertial training has been shown to positively affect muscular strength and sports performance (e.g. agility). However, implementing such eccentrically-biased training during a microcycle needs to be carefully planned due to its purported effects on the neuromuscular system that can last for hours/days post-exercise. This study aimed at using tensiomyography to verify the effects of different inertias during the hip extension exercise on the contractile function of biceps femoris and semitendinosus muscles of the dominant leg for up to 72 hours post-exercise. Thirty participants (24.4 ± 3.4 years) were divided into 0.075 or 0.1 kg·m^2^ inertia groups and a control group. Magnitude-based analysis was used for the comparisons. Several tensiomyography parameters were changed after both intensities of flywheel exercise (in most cases indicating a decrement in muscle stiffness), whereas most between-group differences suggested that in the semitendinosus muscle, the higher inertia (0.1 kg·m^2^) influenced the muscle stiffness parameters more (e.g. Dm = maximal radial displacement) while in the biceps femoris, the greater effect was caused by the lower inertia (0.075 kg·m^2^) (e.g. Tc = contraction time). Most changes in contractile properties of the investigated muscles occur within 24 hours post-exercise, but can persist for up to 72 hours. However, higher inertia (0.1 kg·m^2^) influenced the stiffness of the semitendinosus muscle more, while in the biceps femoris, the greater effect was caused by the lower inertia (0.075 kg·m^2^). These findings should be considered by practitioners when prescribing flywheel iso-inertial training.

## Introduction

Several investigations to date have revealed that eccentric (ECC) training has superior outcomes compared to concentric (CON) training in the development of both muscle hypertrophy and strength [1]. Compared to CON actions, isolated ECC actions are characterized by producing higher torque [2] with lower muscle activation [3] and electromechanical delay [4]. Additionally, ECC actions require lower metabolic cost compared with CON actions [5], as well as higher solicitation of Type IIx fibres [6]. In addition, ECC actions can optimize the efficacy of training (i.e. better adaptation in less time) of elite sports athletes [1, 7]. On the other hand, although ECC-based training has many advantages over CON training, it has been known for 40 years that this type of training produces swelling and a decreased range of motion, being related to changes in passive and active muscle stiffness, as well as a pronounced loss of strength between 24 and 72 hours after exercising [8, 9].

A wide range of ECC paradigms [10, 11] has been developed to focus on ECC actions, among which flywheel iso-inertial resistance training (FLY-RT) has been highlighted, since it shows benefits in both performance and clinical settings [1, 7, 11]. In this line, and in spite of the fact that the importance of the type of inertia for the prescription of training has been demonstrated, there is only one work in the literature analysing the impact of different inertias (i.e. loading stimulus) on performance [12]. These authors employed six different inertias (0.0125–0.1 kg·m^2^) and investigated their effect on power, work, force and eccentric overload. They concluded that athletes looking for power adaptations in concentric and eccentric muscle contraction may use lower inertias, calling for a shorter ECC–CON coupling time and greater power production, whereas practitioners pursuing greater work output during resistance exercise (RE) should employ higher inertias. Considering the benefits of this paradigm, many authors have explored the effects of FLY-RT on hamstring hypertrophy and strength, since hamstring injuries are the most common musculoskeletal injury in both individual and team sports [13]. Two recent investigations [1, 14] employed FLY-RT (inertia of 0.072 kg·m^2^) and demonstrated superior individual muscle use in hamstrings observed by magnetic resonance imaging (MRI) against well-known paradigms to strengthen the hamstrings, such as the Nordic hamstrings or the Russian belt.

In acute observations, the effects of different inertial loads on muscle contractility are unknown; this should be addressed, because an optimal contractile capacity is crucial for subsequent training quality and performance [15]. To this end, a relatively new mechanomyographic method to monitor changes in muscle contractile function named tensiomyography (TMG) has been developed [16]. This apparatus allows evaluation of the changes in muscle contractile properties related to fluctuations in passive muscle stiffness due to loss of muscle tension [17, 18] and fatigue after training sessions [19–22] or competitions [23, 24]. In this sense, accumulated fatigue has been shown to decrease muscle tension and increase passive muscle stiffness [25], delaying the return of elastic energy and deteriorating muscle contractile properties [19, 21, 22, 24, 26].

We hypothesize that passive muscle belly stiffness of the hamstrings will increase with both moderate and high FLY-RT loads, while the contractile capacity is expected to suffer detrimental changes immediately after the intervention but to recover completely after four days. Thus, the main purpose of this study was to analyse power, work and eccentric overload generated during a hip extension executed on a flywheel inertial device with two different inertias in physically active participants and contractile properties post-exercise using TMG-derived variables.

## Materials and methods

### Subjects

Thirty participants (24.4 ± 3.4 years) with no previous hamstring injury, at least in the last year, and three years of experience in strength training participated in the study (Table 1). Previous experience with flywheel inertial devices was not required. Subjects were healthy and moderately active individuals, engaged in two or three strength training sessions per week (> 150 mins). All the participants were informed of the objectives of the research, participated voluntarily, and were able to withdraw from the investigation at any time without penalty. All the participants signed a written informed consent. The study was conducted according to the Declaration of Helsinki, and the protocol was fully approved by the Ethics Committee of the University of Vigo.

**Table 1:** Individual physical characteristics of control and experimental groups. Data: Mean ± SD.

	Age (years)	Height (cm)	Body mass (kg)
**Control group**	24.40 ± 3.44	180 ± 0.05	75.02 ± 2.72
**Group 0.1 kg·m**^**2**^	27.70 ± 3.47	184 ± 0.06	78.98 ± 6.35
**Group 0.075 kg·m**^**2**^	27.20 ± 3.05	183 ± 0.05	77.95 ± 4.00

### Experimental procedures

**Fig 1:**
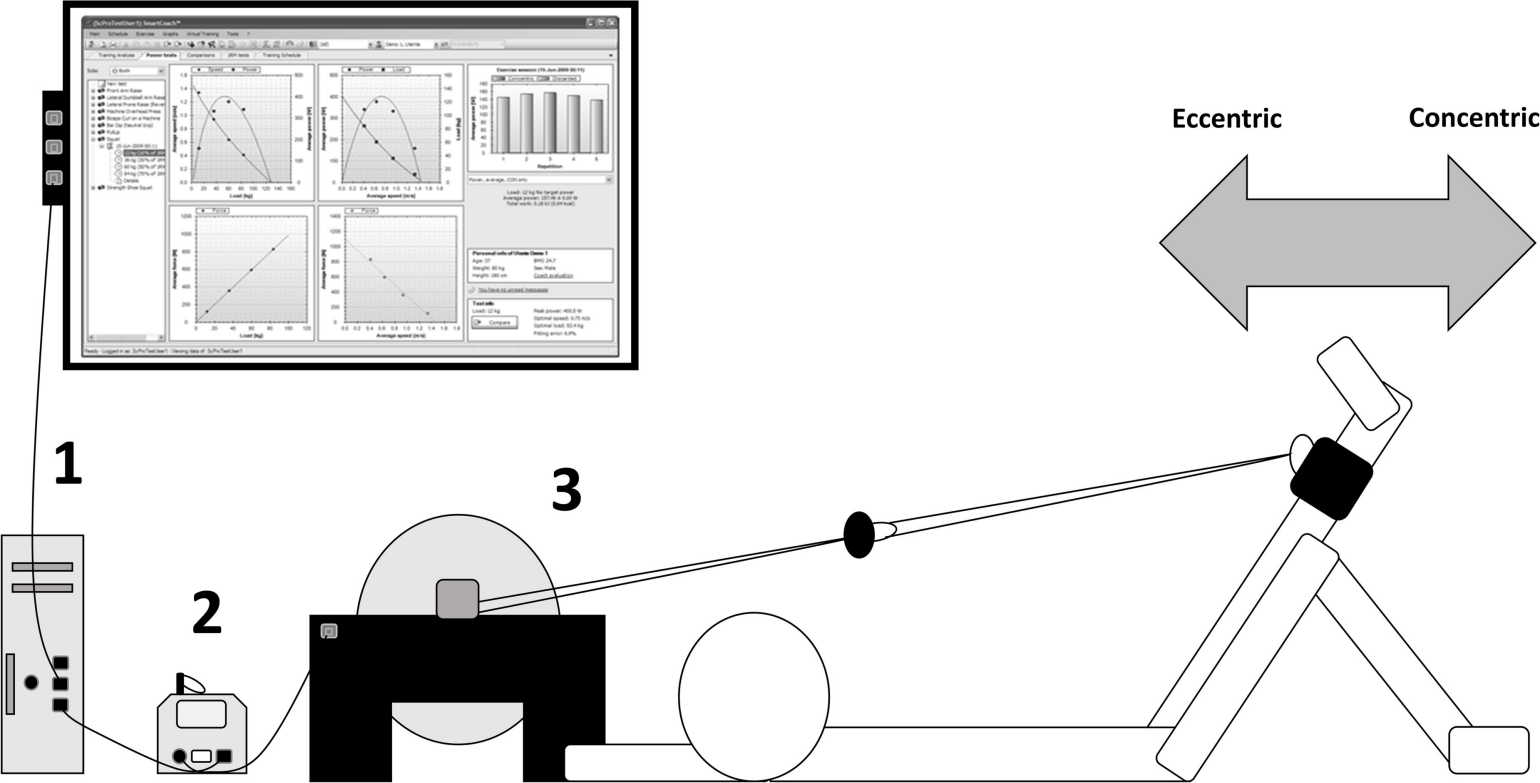
Schematic representation of the hip extension protocol. 1. Software SmartCoach; 2. Sensor SmartCoach; 3. Kbox3.

A repeated measures experimental design was used. All participants (n = 30) were randomly divided into one control (n = 10) and two experimental groups (n = 10 per group) with a specific inertia randomly assigned (0.075 or 0.1 kg·m^2^). Participants performed maximal unilateral coupled CON-ECC hip extensions using an iso-inertial device “Fig 1”. Two familiarization sessions were completed before the inertial test to ensure appropriate technique. A standardized warm-up was completed in all sessions. The protocol was designed with a focus on repeat power ability and was the same for both experimental groups, each with the respective inertia assigned. The control group did not perform any exercise during the intervention period. Mean-peak CON-ECC power and mean CON-ECC velocity during, and perceived exertion and TMG variables post-exercise, were measured. Verbal encouragement was provided during all the flywheel sessions.

**Fig 2:**
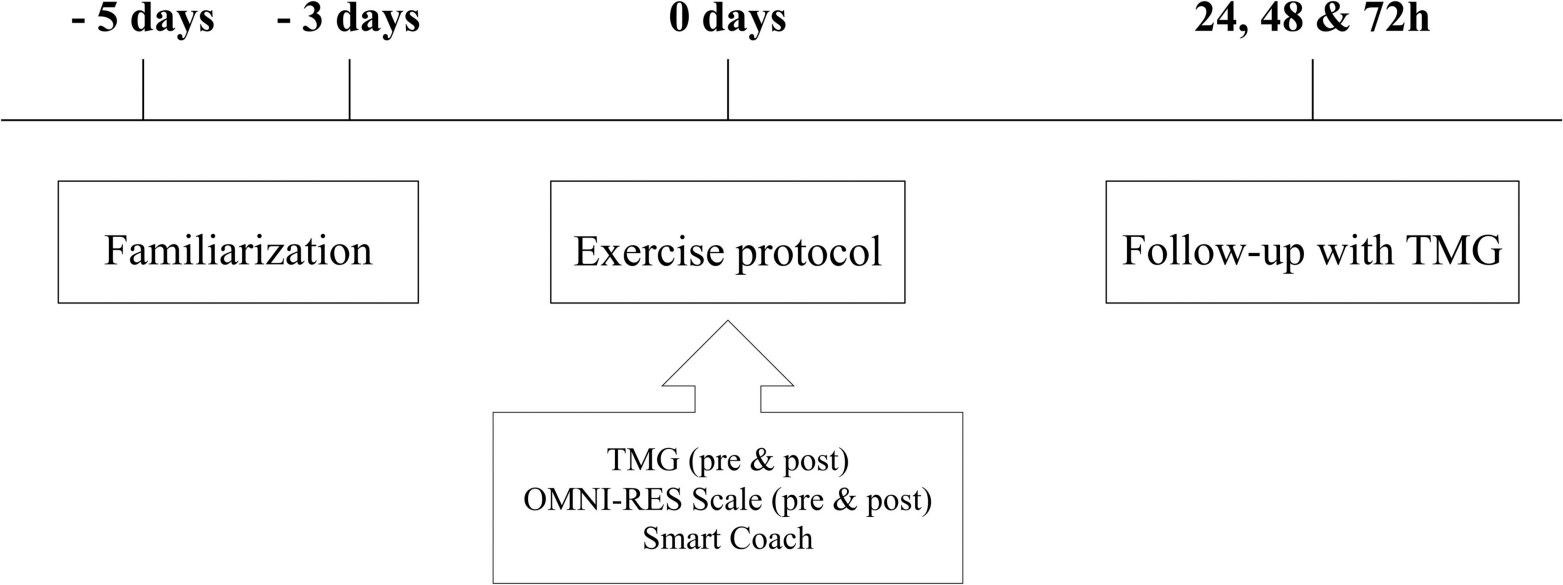
Schematic representation of the protocol. The specific test protocol focused on repeat power ability and consisted of four sets of seven (plus two repetitions to accelerate the inertia) unilateral coupled CON-ECC all-out hip extensions, with 30 seconds’ rest between sets (Table 2).

Before any test using the inertial device, participants performed two familiarization sessions (five and three days before the lower limb power test) to ensure correct technical performance in the subsequent experimental sessions. Moreover, the complete protocol and specific recommendations were explained in detail. Warm-up was standardized for all sessions and consisted of five minutes’ cycling at 80W–80rpm (Ergoline 100, Ergometrics, Bitz, Germany), exercise activation for gluteus maximus, Russian belt (five repetitions), stretching activation, and four repetitions at 70% of maximal measured intensity for each group with the iso-inertial device. The two experimental groups followed the same timeline and protocol; the only difference was the inertia assigned for testing “Fig 2”.

**Table 2:** Training protocols. Data: mean ± SD.

Protocol	Hip extension single leg 0.100 kg·m^2^	Hip extension single leg 0.075 kg·m^2^
**Set x reps**	4 x 7 (+2 acceleration reps)	
**Intensity**	All-out	
**Repetition time (s)**	ECC/CON	
**Total TUT (s)**	125´	130´
**Rest between sets (s)**	30´	
**Displacement (m)**	2.21 ± 0.04	2.22 ± 0.05
**Total action time (s)**	4.48 ± 0.32	4.63 ± 0.13
**Time CON (s)**	2.31 ± 0.16	2.36 ± 0.07
**Time ECC (s)**	2.17 ± 0.17	2.27 ± 0.06

Abbreviations: Reps = maximum repetitions; TUT = time under tension; ECC = eccentric phase; CON = concentric phase; All-out = maximum effort.

Forty-eight hours before each testing session, participants did not perform any strenuous exercise and consumed no stimulant drinks. Moderate and high inertial loads (0.075 to 0.1 kg·m^2^) were chosen because Sabido et al. [27] showed that using these inertial loads achieved the higher phase eccentric-concentric ratio. Participants performed the proposed exercise lying supine on a mat with the strap placed around the ankle. The CON hip extension was done during the descending phase while accelerating the pulley (inertia 0.075 or 0.1 kg·m^2^); the ECC hip extension was done during the ascending phase while decelerating the pulley [1]. Core muscle activation was emphasized during the exercise, and the free leg was fixed to not rise (Fig 1). According to the study by Méndez et al. [1], the hip extension conic-pulley exercise causes an activation in the femoral biceps long head at medial level pre 37.7° ± 4.8, post 40.2° ± 4.0, being a difference of 6%, while in the semitendinosus muscle the activation is pre 36.8 ± 3.5, post 41.1 ± 3.4, being a difference of 11%.

Concentric mean power (CMP), eccentric mean power (EMP), concentric peak power (CPP), eccentric peak power (EPP), mean concentric velocity (MCV) and mean eccentric velocity (MEV) were measured during the lower limb power test with a specific data acquisition device. Also, TMG measurements were performed before the warm-up, just after the lower limb repeat power test, and after 24, 48 and 72 hours. In order to ensure the reliability of the TMG assessment, two pre-test, post-test, and after 24, 48 and 72 hours measurements were taken from each participant. The evaluated muscles were the biceps femoris (BF) and semitendinosus (ST) of the dominant leg. Finally, perceived exertion was obtained through a specific visual assessment scale.

## Equipment and tests

### Iso-inertial device and rotary encoder

A non-gravity dependent iso-inertial device was used to perform the tests (Kbox3 Exxentric AB, Sweden). Devices equipped with iso-inertial technology provide an iso-inertial resistance during coupled CON-ECC actions [11]. The load is given by the inertia of a rotating mass (flywheel), which in turn depends on its geometrical (diameter, thickness) and physical (density of the material) properties. The overall inertia is adjusted through the total number of flywheels installed.

Power-related variables were obtained using a specific analysis feature in a performance-measurement system compatible with flywheel devices (SmartCoach Power Encoder, SmartCoach Europe AB, Stockholm, Sweden), with associated SmartCoach software (v 5.0.0.19). The sampling frequency of the equipment was set to 100 Hz and, based on the inertia and on the average rotational velocity, mean and peak concentric and eccentric power outputs were calculated.

### TMG specifications

TMG was used to assess the mechanical properties of both ST and BF of the dominant leg. Measurements were taken under static and relaxed conditions. Prior to performing the measurements, an accurate digital displacement-transducer (GK 40, Panoptik doo, Ljubljana, Slovenia) was positioned perpendicularly at the highest point of the muscle belly following Perotto et al. [28] for BF, at the midpoint of a line between the fibula head and the ischial tuberosity, and for ST, midway on a line between the medial epicondyle of the femur and the ischial tuberosity. To assure the same placement of electrodes between the consecutive measurements, the point was marked with a permanent skin marker. To elicit the twitch responses, quadrangular adhesive electrodes (5x5cm) (TheraTrode, TheraSigma, California, United States of America) were connected to an electric stimulator (TMG-S1 doo, Ljubljana, Slovenia) and positioned on the muscle surface, following the arrangement of the fibres. The electrodes were placed symmetrically approximately 5 cm away from the sensor, the positive electrode in the proximal area of the muscle above the measurement point and the negative electrode in the distal area below the measurement point, according to previous investigations [29]. Both ST and BF were measured with the participant in the prone position and the knee joint fixed at an angle of ~30° by means of a wedge cushion. Electrical stimulation was applied with pulse duration of 1 ms and initial current amplitude of 30 mA, which was progressively increased in 10 mA steps up to the stimulator’s maximal output (100 mA). A 15-s rest period was allowed between each electrical stimulus to avoid fatigue or post-tetanic activation. All measurements were taken by the same experienced evaluator and only the curve with the highest Dm value was considered for further analysis.

**Fig 3:**
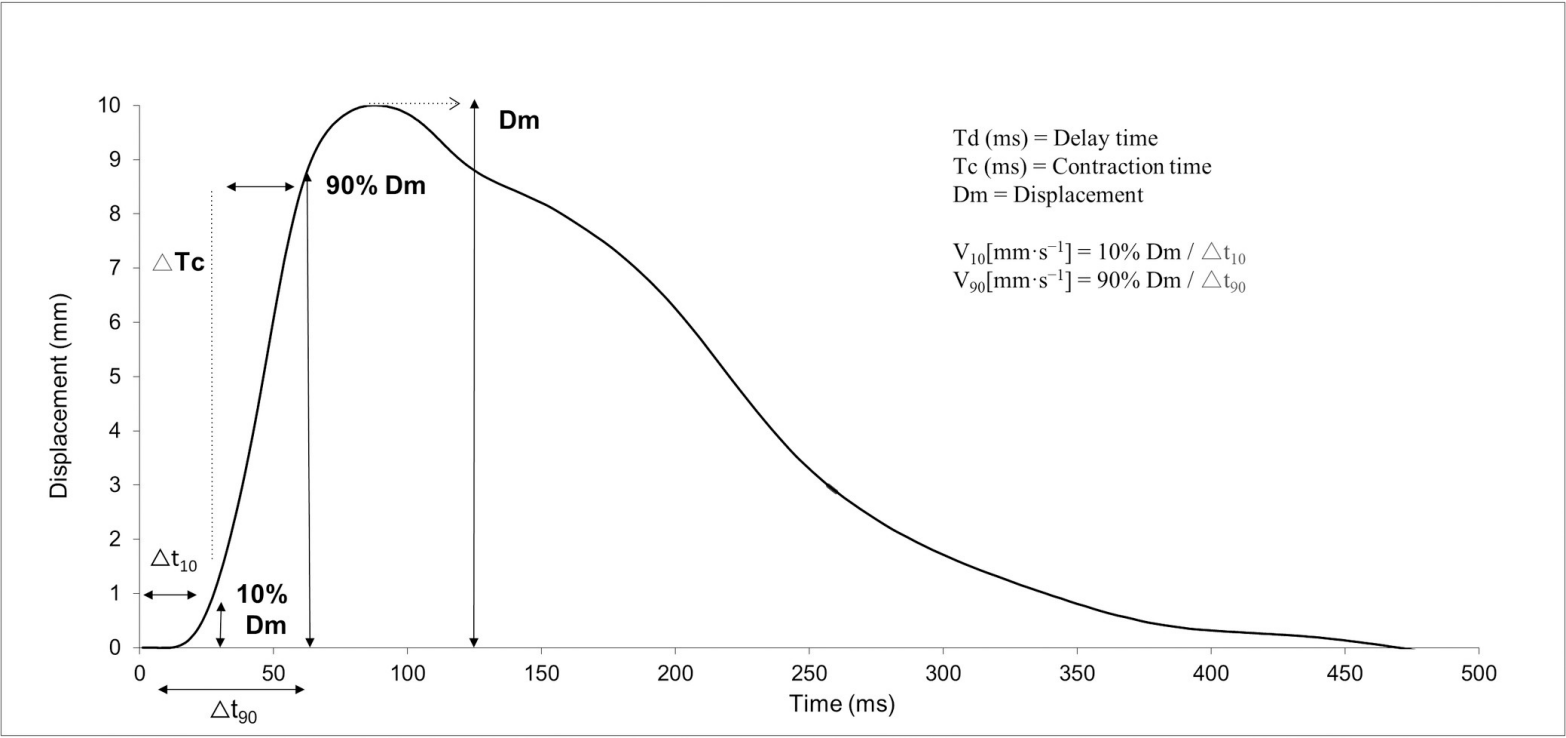
TMG Parameters. Td: delay time; Tc: contraction time; Dm: maximal radial displacement.

Each measurement involved recording the following parameters: maximal radial displacement (Dm) as the peak spatial transverse deformation of skeletal muscle in millimetres (mm), registered by the digital transducer; contraction time (Tc) as the time in milliseconds (ms) from 10% to 90% of Dm on the ascending curve; and delay time (Td) as the time in ms from onset to 10% of Dm. Mean velocities of muscle contraction (mm/s) from the onset of electrical stimulation until 10% (V10) and 90% (V90) of Dm were also recorded “Fig 3”. Both velocities of contraction were calculated following the equations developed by de Paula Simola et al. [19].

### Perceived exertion scale

The OMNI-RES scale was developed for use in resistance exercise in a specific way [30]. It is based on a pictorial format located above an ascending numerical range of 0–10 (0 = extremely easy and 10 = extremely hard). Verbal descriptors located at the bottom correspond to specific numerical and pictorial categories. The subjects were familiarized with the OMNI-RES scale during the two prior sessions and were asked to select a scale value to assess their effort level 30 minutes after exercise.

### Statistical analysis

Data are presented as mean ± standard deviation. All data were log-transformed before analysis to reduce bias arising from non-uniformity error and then analysed for practical significance using magnitude-based inferences [31]. Intrarater reliability of TMG parameters was calculated through intraclass correlation coefficient reliability (ICCR) analysis by a two-way mixed effects model and absolute agreement. For within-group comparisons, the percentage change and 90% confidence interval (90% CI) of the muscles’ contractile properties were expressed and compared in relation to measurements carried out before iso-inertial exercise. Standardized difference or effect size (Cohen’s d coefficient) and 90% CI were calculated for between-group comparisons of the effect of the iso-inertial exercise on muscles’ contractile proprieties. The magnitudes of the Cohen’s d values were qualitatively interpreted using the following thresholds: < 0.2 trivial; 0.2–0.6 small; 0.6–1.2 moderate; 1.2–2.0 large; 2.0–4.0 very large; and 4.0 extremely large [31]. The quantitative chances of finding within- and between-group differences were calculated and inferred qualitatively as follows: 1% almost certainly not; 1% to 5% very unlikely; 5% to 25% unlikely; 25% to 75% possible; 75% to 95% likely; 95% to 99% very likely; 99% almost certain. If the chances of having lower and higher results were both > 5%, the true difference was rated as unclear [31]. The statistical analyses were performed using an Excel spreadsheet (http://www.sportsci.org).

## Results

### Iso-inertial device and rotary encoder

There were unclear differences between the groups for CMP, EMP, CPP and EPP across the four iso-inertial exercise sets. However MCV and MEV were likely to very likely higher (moderate effect size) in the 0.075 group. In addition, the OMNI-RES was likely higher (moderate effect size) in the 0.1 group (Table 3).

**Table 3:** Comparison of the power and velocity output during iso-inertial exercise in the 0.1 kg·m2 and 0.075 kg·m^2^ groups.

	Group 0.1 kg·m^2^			Group 0.075 kg·m^2^			ES (CL90%)		%Chances (+/trivial/-)	Outcome
**CMP, Watts**										
**1**^**st**^ **Set**	121.6	±	25.4	123.8	±	19.6	-0.13	(-0.88; 0.61)	22/34/44	Unclear
**2**^**nd**^ **Set**	115.0	±	30.2	114.7	±	19.7	-0.06	(-0.81; 0.68)	27/35/38	Unclear
**3**^**rd**^ **Set**	108.6	±	29.0	107.5	±	17.8	-0.06	(-0.80; 0.69)	28/35/37	Unclear
**4**^**th**^ **Set**	100.6	±	28.6	100.3	±	14.3	-0.11	(-0.86; 0.64)	24/45/32	Unclear
**EMP, Watts**										
**1**^**st**^ **Set**	107.0	±	18.0	102.1	±	20.2	0.25	(-0.50; 0.99)	54/30/16	Unclear
**2**^**nd**^ **Set**	96.1	±	18.7	96.4	±	17.3	-0.06	(-0.80; 0.69)	28/35/37	Unclear
**3**^**rd**^ **Set**	90.8	±	24.0	91.8	±	15.7	-0.13	(-0.88; 0.61)	22/34/44	Unclear
**4**^**th**^ **Set**	80.3	±	23.0	86.1	±	14.8	-0.37	(-1.13; 0.37)	10/25/65	Unclear
**CPP, Watts**										
**1**^**st**^ **Set**	220.4	±	45.1	210.3	±	31.0	0.21	(-0.54; 0.95)	51/32/18	Unclear
**2**^**nd**^ **Set**	208.7	±	48.2	199.9	±	33.4	0.16	(-0.59; 0.90)	46/33/21	Unclear
**3**^**rd**^ **Set**	194.7	±	53.7	187.3	±	33.9	0.09	(-0.66; 0.83)	40/35/26	Unclear
**4**^**th**^ **Set**	174.2	±	56.5	169.3	±	28.2	0.00	(-0.75; 0.75)	32/36/32	Unclear
**CEP, Watts**										
**1**^**st**^ **Set**	196.4	±	35.4	190.9	±	36.2	0.17	(-0.58; 0.91)	47/33/20	Unclear
**2**^**nd**^ **Set**	183.8	±	35.4	171.1	±	30.9	0.36	(-0.39; 1.10)	64/25/11	Unclear
**3**^**rd**^ **Set**	171.7	±	37.7	161.6	±	29.3	0.26	(-0.48; 1.01)	56/30/15	Unclear
**4**^**th**^ **Set**	155.6	±	44.0	149.5	±	27.1	0.10	(-0.65; 0.84)	41/25/35	Unclear
**MCV, laps·s**^**-1**^										
**1**^**st**^ **Set**	6.9	±	0.5	7.3	±	0.5	-0.90	(-1.65; -0.16)	01/05/94	***Likely***
**2**^**nd**^ **Set**	6.6	±	0.5	7.1	±	0.4	-0.95	(-1.70; -0.21)	01/04/95	***Very Likely***
**3**^**rd**^ **Set**	6.4	±	0.5	6.8	±	0.5	-0.66	(-1.40; 0.09)	03/12/85	***Likely***
**4**^**th**^ **Set**	6.1	±	0.5	6.7	±	0.5	-0.99	(-1.74; -0.25)	01/03/96	***Very Likely***
**MEV, laps·s**^**-1**^										
**1**^**st**^ **Set**	6.7	±	0.6	7.1	±	0.6	-0.55	(-1.31; 0.21)	5/16/78	Unclear
**2**^**nd**^ **Set**	6.4	±	0.5	6.9	±	0.5	-0.91	(-1.66; -0.17)	01/05/94	***Likely***
**3**^**rd**^ **Set**	6.2	±	0.6	6.7	±	0.5	-0.85	(-1.60; 0.11)	01/06/93	***Likely***
**4**^**th**^ **Set**	6.0	±	0.5	6.5	±	0.5	-1.01	(-1.75; -0.26)	01/03/96	***Very Likely***
**OMNI-RES**	8.2	±	1.6	7.2	±	1.7	0.63	(-0.11; 1.38)	84/13/03	***Likely***

Abbreviations: CMP: concentric mean power; EMP: eccentric mean power; CPP: concentric peak power; EPP: eccentric peak power; MCV: mean concentric velocity; MEV: mean eccentric velocity; OMNI-RES: OMNI perceived exertion scale for resistance exercise; ES (90%CI): effect size (90% confidence interval); %Chances (+/trivial/-): percentage chances of the real value being higher, trivial or lower in the 0.1 group than the 0.075 group; Chances higher than 75% are highlighted in grey

### TMG

#### Reliability

The contraction time ICC values obtained were 0.97 (CI 0.92–0.99) for Tc before; 0.99 (CI 0.96–0.99) for Tc immediately post; 0.99 (CI 0.93–0.99) for Tc 24h post; 0.99 (CI 0.96–0.99) for Tc 48h post; and 0.99 (CI 0.97–0.99) for Tc 72h post. The maximum radial displacement ICC values were 0.96 (CI 0.89–0.98) for Dm before; 0.98 (CI 0.95–0.99) for Dm immediately post; 0.98 (CI 0.77–0.99) for Dm 24h post; 0.99 (CI 0.96–0.99) for Dm 48h post; and 0.99 (CI 0.97–0.99) for Dm 72h post.

#### Within-group comparisons

**Fig 4:**
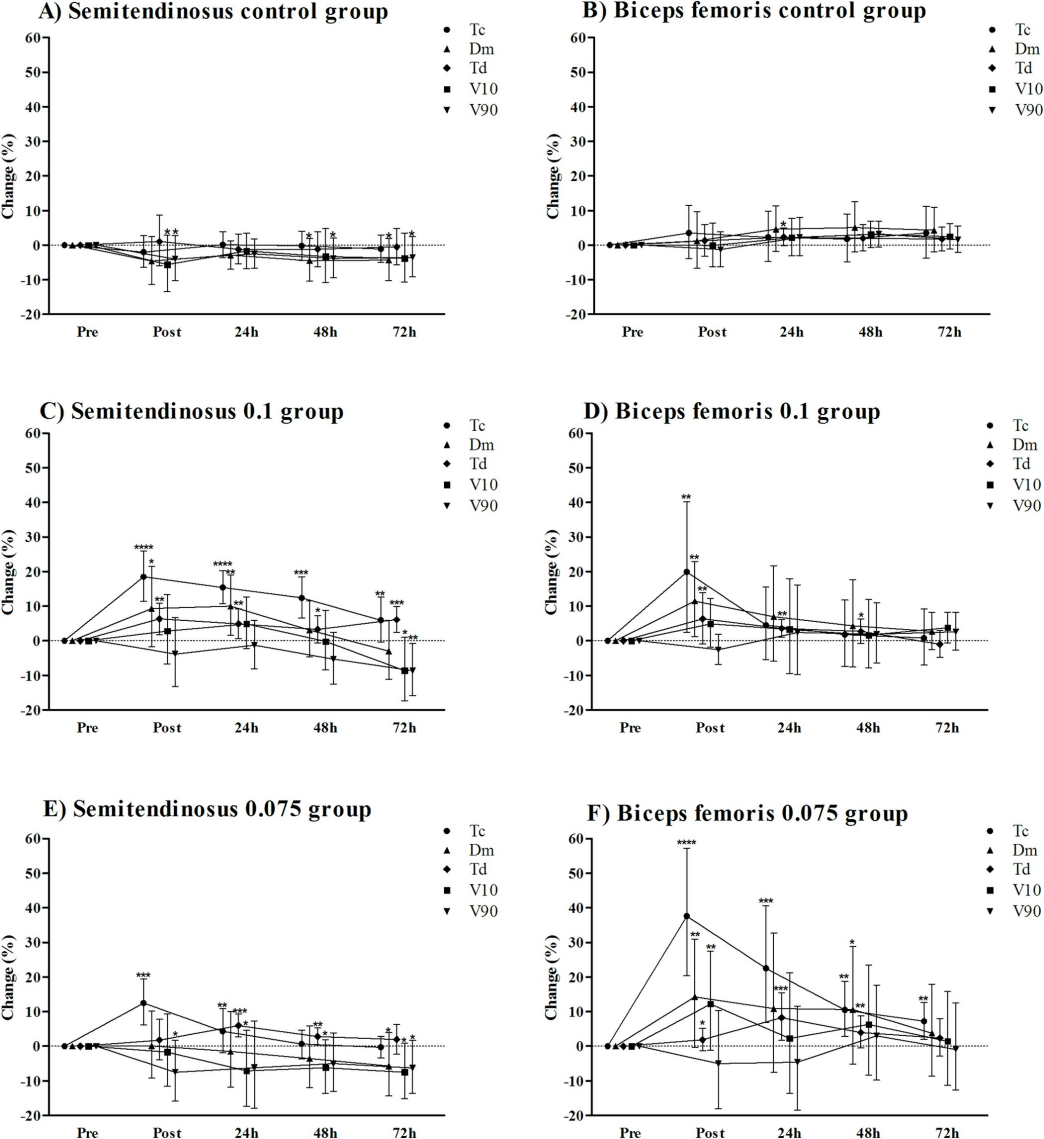
Muscle contractile properties changes immediately, 24h, 48h and 72h after control condition and iso-inertial exercise. Panel A: semitendinosus muscle of the control group; Panel B: biceps femoris muscle of the control group; Panel C: semitendinosus muscle of the 0.01 group; Panel D: biceps femoris muscle of the 0.1 group; Panel E: semitendinosus muscle of the 0.075 group; Panel F: biceps femoris muscle of the 0.075 group. Data are mean and 90% confidence interval. Tc: contraction time; Dm: maximal radial displacement of the muscle belly; Td: delay time; V_10_: muscle contraction velocity at 10% Dm; V_90_: muscle contraction velocity at 90% Dm. ****almost certainly, ***very likely, **likely, *possibly differences in relation to baseline (pre) measurement.

The contractile properties of the ST and BF muscles measured before, immediately post and 24, 48 and 72 hours after control condition (control group) or iso-inertial exercise (0.1 and 0.075 groups) are shown in “Fig 4”.

There were no meaningful changes in the contractile proprieties of the ST and BF muscles in the control group. In the 0.1 group, the Tc, Dm and Td of the ST muscle possibly to almost certainly increased (change ranged from 3.3% to 18.5%) throughout 72 hours, whereas V_10_ possibly and V_90_ likely decreased (both -8.6%) only in the measurement obtained 72 hours after iso-inertial exercise. In the BF muscle, the Tc, Dm and Td likely increased (change ranged from 2.7% to 19.9%) immediately after iso-inertial exercise. However, only the Td remained likely and possibly increased after 24 (3.6%) and 48 hours (2.7%) respectively. In the 0.075 group, the Tc of the ST muscle very likely increased immediately after iso-inertial exercise (12.5%) and remained likely increased after 24 hours (4.3%). The Dm possibly decreased only 72 hours (-5.7%) after iso-inertial exercise, while Td very likely and likely increased only after 24 (6.0%) and 48 hours (2.8%) respectively. The V_10_ possibly decreased 24 (-7.1%), 48 (-6.2%) and 72 hours (-7.5%) after iso-inertial exercise; V_90_ possibly decreased immediately (-7.5%) and 72 hours (-6.3%) after iso-inertial exercise. In the BF muscle, the Tc likely to almost certainly increased (change ranged from 7.2% to 37.6%) throughout 72 hours. The Dm likely increased immediately (14.3%) and possibly increased 48 hours (10.6%) after iso-inertial exercise. The Td possibly to very likely increased (change ranged from 1.9% to 8.3%) throughout 48 hours. The V_10_ likely increased only in the measurement obtained immediately (12.2%) after iso-inertial exercise.

#### Between-group comparisons

**Fig 5:**
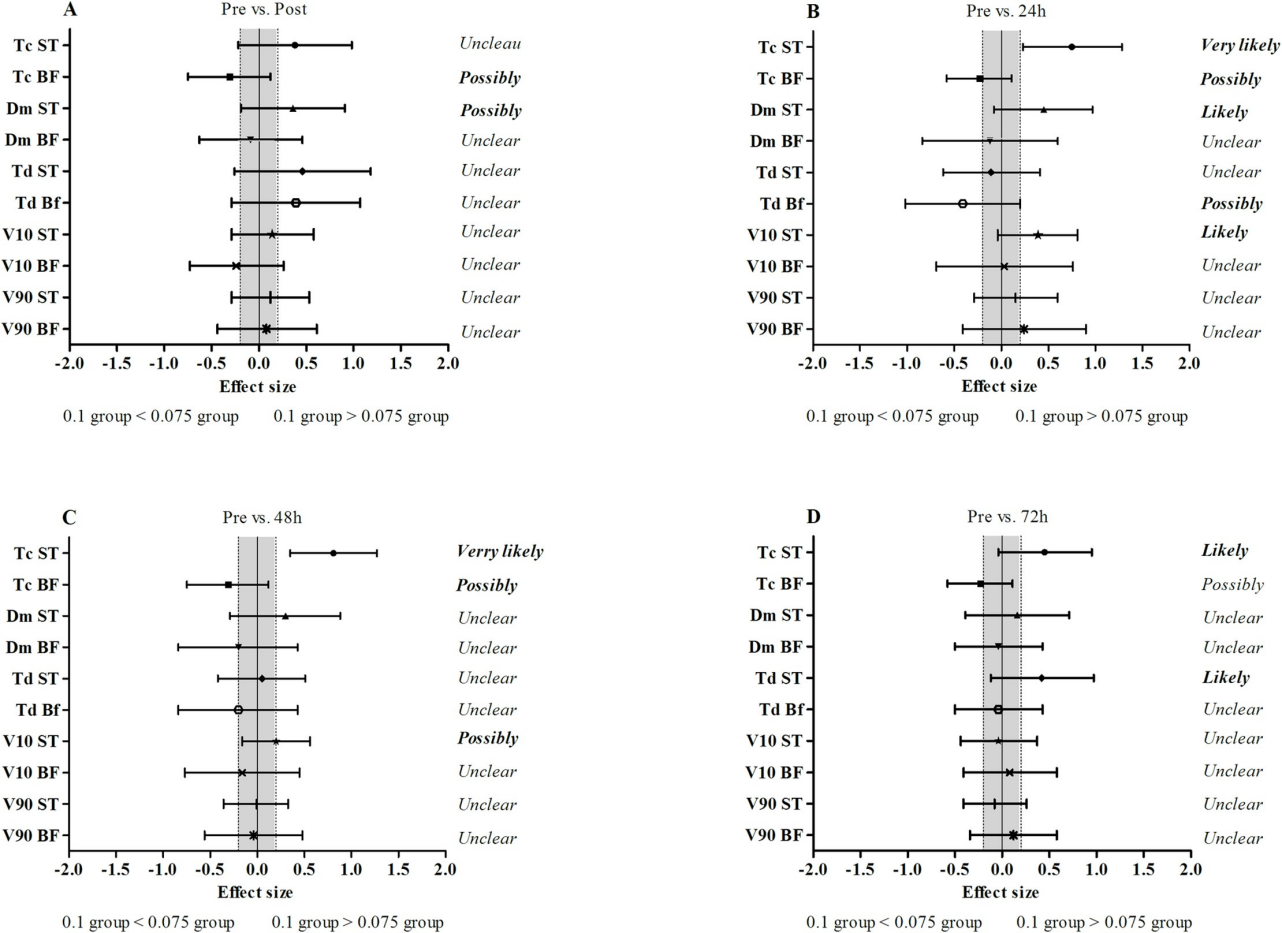
Standardized difference (effect size) between 0.01 and 0.075 groups in the muscle contractile proprieties changes immediately, 24h, 48h and 72h after iso-inertial exercise. Panel A: Pre- vs. Post; Panel B: Pre- vs 24h; Panel C: Pre- vs. 48h; Panel D: Pre- vs. 72h. Bars are 90% confidence interval. Grey area represents the smallest worthwhile difference (0.2 based on Cohen’s principle). ST: semitendinosus muscle; BF: biceps femoris muscle. Tc: contraction time; Dm: maximal radial displacement of the muscle belly; Td: delay time; V_10_: muscle contraction velocity at 10% Dm; V_90_: muscle contraction velocity at 90% Dm.

The comparisons between the changes in experimental groups (0.1 vs. 0.075) for muscle contractile properties immediately, 24, 48 and 72 hours after iso-inertial exercise are shown in “Fig 5”. Immediately after the iso-inertial exercise, the effect on Tc of the BF muscle was possibly higher in the 0.075 group (19.9% vs. 37.6%), whereas the effect on Dm of the ST muscle was possibly higher in the 0.1 group (9.3% vs. 0.1%). A likely to very likely higher effect was observed 24 hours after iso-inertial exercise for the 0.1 group in the Tc (15.4% vs. 4.3%), Dm (10.0% vs. -1.5%) and V_10_ (4.9% vs. -7.1%) of the ST muscle, whereas the effect on Tc in the BF muscle was possibly higher for the 0.075 group (4.5% vs. 22.5%). A very likely and possibly higher effect was observed 48 hours after iso-inertial exercise in the 0.1 group for Tc (12.4% vs. 0.7%) and V_10_ (-0.2% vs. 6.2%) of the ST muscle, whereas the effect on Tc of the BF muscle was possibly higher for the 0.075 group (1.8% vs. 10.5%). Seventy-two hours after iso-inertial exercise, a likely higher effect was observed in the 0.1 group only for Tc (5.9% vs. -0.3%) and Td (6.1% vs. 2.0%) of the ST muscle.

## Discussion

Our hypothesis in this study was that passive muscle belly stiffness of the hamstrings would increase with both moderate and high FLY-RT loads. However, this seems not to be confirmed.

Notably, the 0.075 kg·m^2^ resulted in higher contraction velocities in both the concentric and eccentric phases, while 0.1 kg·m^2^ resulted in higher rating of perceived exertion. Despite this, no differences in mean and maximal concentric and eccentric power were noted between different inertias. In the within-group analyses, several TMG parameters were changed after both intensities of flywheel exercise (in most cases, indicating a decrement in muscle stiffness), whereas most between-group differences were found in < 72h, suggesting that in the ST, the higher inertia (0.1 kg·m^2^) influenced the muscle stiffness parameters more, while in the BF, the greater effect was caused by the lower inertia (0.075 kg·m^2^).

In this study, the performance measurement device did not reveal differences related to power production (i.e. CMP, EMP, CPP and CEP) between 0.1 and 0.075 kg·m^2^ loads. This was somewhat surprising, since it was previously shown that in both men and women the same increase in the inertial load (0.025 kg·m^2^) using the knee extension flywheel led to a significant decrement in the concentric power [1]. However, it should be noted that in our study we did not use a within-subject design in the comparison between different loads. This may have biased our results. Nevertheless, it appears that when performing with lower loads, participants increased their velocity of contraction, thus maintaining the power output. This may be an important compensatory effect that needs to be further investigated. It is of note that the 0.1 group displayed higher OMNI-RES levels; this is consistent with the fact that perception of effort is modulated by the central commands to the skeletal muscles during exercise [32]. In this sense, higher loads probably drive higher amplitudes of movement-related cortical potential and thus higher rating of perceived exertion [32].

If one considers a value below 0.8 as the cut-off for insufficient reliability [33], then the ICCs for our data indicate good reliability for all the TMG variables. In fact, some of them (Dm, Tc and Td) yielded very high reliability coefficients. TMG measurements of the BF and ST have previously been reported to show good same-day reliability [34]. This study therefore confirms previous reports.

Markers of skeletal muscle contractile properties (Dm, V_10_ and V_90_) of the lower extremity (BF and ST) were determined using the non-invasive TMG method. The main TMG parameter (Dm) has been proposed as a measure of muscle belly stiffness, having its origin in a mechanomyographic (MMG) study by Evetovich et al. [17]. Relations between Dm and stiffness were subsequently found in the studies by Pisot et al. [18] and Giovanelli et al. [24]. In their study, Evetovich et al. [17] aimed to evaluate the effect of velocity of contraction on the MMG responses to maximal concentric isokinetic leg extension movements. These authors found that there was a velocity-related dissociation between MMG and peak torque—i.e. increased velocity of contraction was associated with increased MMG amplitude (i.e. Dm TMG) but with decreased peak torque. Their results are in agreement with previous investigations of the MMG-peak torque relationship, which have demonstrated that, as the velocity of contraction increases, peak torque decreases because fewer muscle fibres, and therefore cross-bridges, are loaded [35]. Their results were confirmed ten years later by Pisot et al. [18] showing a high decrease in muscle thickness (~15%) of several muscles of the thigh and leg after 35 days of horizontal bed rest, which was significantly correlated (r = -0.70, p < 0.01) with increased Dm (~30%) of the gastrocnemius medialis. To further strengthen and confirm the relationship between Dm and changes in muscle stiffness, Giovanelli et al. [24] observed that vastus lateralis Dm increased by 24% after an uphill marathon (43 km, 3063m elevation gain), while the other investigated temporal TMG parameters decreased (Tc, Td, Tr and Ts), suggesting that the vastus lateralis muscle was less stiff and reacted faster to the electrical stimulus.

Together with TMG changes, Giovanelli et al. [24] observed that the maximal mechanical power of lower limbs significantly decreased after the marathon; vertical stiffness (K_vert_) and leg stiffness (K_leg_) significantly decreased too, demonstrating a decrease in peak torque, in agreement with the findings of Evetovich et al. [17], which were further confirmed by Pisot et al. [18] through the relationship found between the increase in TMG amplitude (Dm) and the decrease in muscle thickness (lower stiffness). In our study, the FLY-RT protocol caused a possible Dm ST increase in the group using the 0.1 (kg·m^2^) load immediately after the exercise and remained likely increased 24 hours later; however, this parameter did not show significant changes after 48 or 72 hours. These results suggest that the stiffness of the ST remained low up to 24 hours after the FLY-RT protocol, but these alterations were not maintained in the follow-up (i.e. 48 or 72 hours later). These changes in the ST’s stiffness were only present in the group with the 0.1 (kg·m^2^) load, not in the 0.075 (kg·m^2^), suggesting that the load played a crucial role in such changes, as reported in several studies [1, 27, 36]. It therefore seems that monitoring the changes in muscle and tendon stiffness is very relevant. In fact, some studies appear to indicate that low or too low levels of muscle/tendon stiffness may allow for excessive joint motion, leading to soft tissue injury [37, 38]. Coaches should therefore keep in mind that stiffness is a modifiable neuromechanical property of tendons and muscles [39, 40]. In this regard, it is in their hands to find strategies to optimize the appropriate levels of stiffness that favour performance and minimize the risks of injury.

At the same time, V_10_ and Tc showed very likely increments in ST after 24 hours indicating, together with Dm changes, that the muscle was impaired at both the neural and the structural level, as previously indicated in some TMG studies [23, 24]. In this regard, temporal parameters remained possibly (V_10_ and Tc) and likely (Tc and Td) elevated 48 and 72 hours respectively after the FLY-RT protocol in the group which used the 0.1 (kg·m^2^) load, indicating that the contraction capacity of the ST muscle was impaired for several days post-exercise. Since the formula for V_10_ is a ratio integrating Dm (10%) as the nominator and Td (total) as the denominator, it can be suggested that Dm to some extent (trends) affected the possibly higher effect in the group which used the 0.1 load (kg·m^2^), but its effect disappeared at 72 hours, only the denominator (Td) possibly remaining elevated. In contrast, the effects of the FLY-RT protocol on the contractile capacity of the BF muscle were only elevated for some temporal parameters (Tc and Td) 24 and 48 hours after the exercise for the group which used the 0.075 (kg·m^2^) load. In this regard, Tc and Td possibly presented increments 24 hours after the exercise, while 48 hours after, only Tc possibly presented increments. These results are unexpected, since the group which used the higher load (0.1 kg·m^2^) did not present significant changes for Dm or any temporal parameter in the BF muscle. Although the participants were randomly distributed, the results obtained in the lower limb power test device showed that there were no differences (p = 0.097) between the group that used the high inertial load (0.1 kg·m^2^) and the group that used the low load (0.075 kg·m^2^) (133.27 ± 16.08 W and 128.05 ± 9.95 W, respectively). Given the above, we suggest that our unexpected results were not due to differences between the two groups, but possibly to a study limitation (lack of *a priori* sample size calculation).

The differences in the changes of the contractile properties between BF and ST can be explained in part by their different muscular composition. Pierrynowski and Morrison [41] showed that BF was composed of 65% slow fibres, 10% fast fibres IIa and 25% fast fibres IIb, while the ST was composed of 50% slow fibres, 15% fast fibres IIa and 35% fast fibres IIb. In this way, it seems reasonable that ST is the muscle that fatigues first and therefore needs more time to recover.

Taken together, these results suggest that the FLY-RT protocol using a high iso-inertial load (0.1 kg·m^2^) causes detrimental changes to the contractile capacity of the hamstring muscles, especially on the ST, which remains impaired until 48 hours after the protocol and, to some extent, even up 72 hours after. Our data also suggest that the lower load (0.075 kg·m^2^) does not produce detrimental changes to the contractile capacity of the ST but produces some non-significant alterations (possibly elevated) in some temporal parameters (Tc and Td) of the BF. Although there is a study that analysed the effect of a FLY-RT protocol [42] on rectus femoris (RF) contractile properties immediately and 24–72 hours after, our study is the first showing disturbances in the hamstrings after FLY-RT. In this regard, our data cannot be compared with the study by de Paula Simola et al. [42], since the muscles evaluated were completely different—i.e. knee flexor (RF) and knee extensors (BF and ST)—although they also found detrimental changes immediately and 24–72 hours after the exercise in some parameters (Dm, V_10_ and V_90_). The previous authors [42] observed the greatest post-exercise decrease in maximal voluntary isometric contraction and the highest lactate production after FLY-RT in post-training, along with changes in TMG parameters (compared to other exercise modes), making a high influence of FLY-RT on neuromuscular disturbance evident. The above agrees with the vast literature showing that ECC muscle actions result in higher tension produced per crossbridge and progressive sarcomere overstretching, predisposing contractile proteins to destruction and damage in cellular structures as sarcolemma, sarcoplasmic reticulum and T-tubules [43].

In spite of the great potential and evidenced benefits derived from the use of iso-inertial devices to develop strength and muscle mass or to prevent injuries [7, 11, 44], coaches must be aware of correctly planning the workloads and in which microcycles to introduce them due to muscle damage [36] and detrimental changes in the contractile properties they cause [36].

Our findings could suggest to coaches that the use of inertial ECC training reduces hamstring stiffness, so coaches can develop strategies to modulate muscle stiffness to comply with their purposes. In addition, higher inertial loads deteriorate ST contractile properties more, while lower inertias affect the BF more. The above finding indicates that coaches should pay special attention to the inertial loads when planning to maintain/increase performance in order to optimize the stimulus and produce the desired training effects. However, this research is limited by some factors, such as the absence of methods to assess stiffness to compare and reaffirm our Dm results, since this is a novel method to indirectly assess muscle stiffness: studies are therefore needed to clarify the relationship between Dm and other types of stiffness measurement (e.g. hop test, Myoton). On the other hand, the absence of maximum voluntary isometric contraction (MVIC) assessment slightly limits the interpretation of TMG parameters.

## Conclusions

In summary, the data presented here indicate that the inertia 0.075 kg·m^2^ resulted in higher contraction velocities in both concentric and eccentric phases, while 0.1 kg·m^2^ resulted in higher rating of perceived exertion. A decrement in muscle stiffness was found after both intensities of flywheel exercise, but the higher inertia (0.1 kg·m^2^) influenced the muscle stiffness of ST more, while in the BF, the greater effect was caused by the lower inertia (0.075 kg·m^2^).
