# Correction: Effects of moderate vs. high iso-inertial loads on power, velocity, work and hamstring contractile function after flywheel resistance exercise

**DOI:** 10.1371/journal.pone.0215567

**Published:** 2019-04-12

**Authors:** Francisco Piqueras-Sanchiz, Saúl Martín-Rodríguez, Luis Manuel Martínez-Aranda, Thiago Ribeiro Lopes, Javier Raya-González, Óscar García-García, Fábio Yuzo Nakamura

There is an error in Table 2. The headings Hip extension single leg 0.100 kg·m^2^ and Hip extension single leg 0.075 kg·m^2^ are swapped in the second and third columns. The second column should be Hip extension single leg 0.075 kg·m^2^ and the third column should be Hip extension single leg 0.100 kg·m^2^. Please see the correct Table 2 here.

**Table 2:** Training protocols.

Protocol	Hip extension single leg 0.075 kg·m^2^	Hip extension single leg 0.100 kg·m^2^
**Set x reps**	4 x 7 (+2 acceleration reps)	
**Intensity**	All-out	
**Repetition time (s)**	ECC/CON	
**Total TUT (s)**	125´	130´
**Rest between sets (s)**	30´	
**Displacement (m)**	2.21 ± 0.04	2.22 ± 0.05
**Total action time (s)**	4.48 ± 0.32	4.63 ± 0.13
**Time CON (s)**	2.31 ± 0.16	2.36 ± 0.07
**Time ECC (s)**	2.17 ± 0.17	2.27 ± 0.06

Abbreviations: Reps = maximum repetitions; TUT = time under tension; ECC = eccentric phase; CON = concentric phase; All-out = maximum effort.
